# 

**Published:** 1803-04-01

**Authors:** 


### To CORRESPONDENTS.

We have again postponed the *Observations* on the Proposal for the radical Cure of Bubonocele, principally because,

1. The chief object of our Journal is to *encourage* and disseminate all improvements connected with Medical science; but these observations are calculated, and apparently designed, to discourage the proposed operation.

2. We wish to give experienced surgeons more time to consider of the proposal in the state in which it is laid before them.

Our readers may perhaps be desirous of knowing on what grounds it is discouraged in the *Observations* abovementioned, and we see no objection to the stating of them.

It is said that the structure of the parts is such, that the complaint will return, or the cure be only temporary. This is but an opinion, and the opinion of the proposers is in direct opposition to it.

It is also said that the operation was many times performed by a Mr. Lee, and never with success; but this Mr. Lee is represented by the writer, as a character of little respectability or professional reputation, and therefore no inference can be drawn from his want of success.

We have received some useful practical observations on the furniture of a sick chamber from Mr. Marson, and beg him to continue them, as we think them well deserving the attention of the public.

We have also received, from our Correspondent at Paris, a most interesting coloured engraving, representing the appearance of sections of the different kinds of Calculi depending on their constituent parts; but it will require a month to get it properly executed.

We are moreover favoured by an excellent Analysis of a most learned and (in Denmark) celebrated work, by Prof. Hensler, on Leprosy, a disease now scarcely known in Europe but by name. We hope the same will soon be said of the Small-pox. This interesting Analysis is delayed on account of the difficulty of procuring suitable types for the Asiatic quotations.

Communications are received from Drs. Mossman and Noehden, Messrs. Ramsay, Okes, Indagator, Medicus, B. G. and a Country Practitioner.

The Editors earnestly request their Correspondents to send them an account of the commencement, continuance, and symptoms of the present Epidemic, and their opinion respecting its contagious nature.

